# The aerobic diagenesis of Mesoproterozoic organic matter

**DOI:** 10.1038/s41598-018-31378-6

**Published:** 2018-09-06

**Authors:** Xiaomei Wang, Wenzhi Zhao, Shuichang Zhang, Huajian Wang, Jin Su, Donald E. Canfield, Emma U. Hammarlund

**Affiliations:** 1 Key Laboratory of Petroleum Geochemistry, Research Institute of Petroleum Exploration and Development, China National Petroleum Corporation, Beijing, 100083 China; 2 Institute of Biology and Nordic Center for Earth Evolution (NordCEE), University of Southern Denmark, Campusvej 55, 5230 Odense M, Denmark

## Abstract

The Xiamaling Formation in the North China Block contains a well-preserved 1400 Ma sedimentary sequence with a low degree of thermal maturity. Previous studies have confirmed the dynamic and complex nature of this evolving marine setting, including the existence of an oxygen-minimum zone, using multi-proxy approaches, including iron speciation, trace metal dynamics, and organic geochemistry. Here, we investigate the prevailing redox conditions during diagenesis via the biomarkers of rearranged hopanes from the finely laminated sediments of the organic-rich black shales in Units 2 and 3 of the Xiamaling Formation. We find that rearranged hopanes are prominent in the biomarker composition of the oxygen-minimum zone sediment, which is completely different from that of the sediment in the overlying anoxic strata. Since the transition process from hopanes to rearranged hopanes requires oxygen via oxidation at the C-l6 alkyl position of 17α(H)-hopanes, we infer that dissolved oxygen led to the transformation of hopane precursors into rearranged hopanes during the early stages of diagenesis. The use of hopanoid hydrocarbons as biomarkers of marine redox conditions has rarely been previously reported, and the hydrocarbon signatures point towards oxic bottom waters during the deposition of Unit 3 of the Xiamaling Formation, which is consistent with the earlier oxygen-minimum zone environmental interpretation of this Unit.

## Introduction

Oxic respiration and aerobic diagenesis dominate modern deep-sea marine sediments due to the high oxygen concentrations in the modern atmosphere and oceans. However, based on geologic evidence, the Mesoproterozoic-era marine environment is generally believed to have been anoxic below the upper surface layer^1–5^. Because of this, it would have been difficult for marine organic matter in Mesoproterozoic sedimentary environments to have undergone decomposition by aerobic diagenesis. The low oxygen levels in the water column are also considered to have restricted the evolution and diversification of eukaryote clades^6^, including animals, until a permissive environment emerged with an increase in oxygen levels in the late Neoproterozoic era^7,8^.

Despite these generalities, the actual levels of Mesoproterozoic atmospheric oxygen and the extent of ocean oxygenation have remained a matter of debate. Thus, the relatively low concentrations of redox-sensitive trace metals in Mesoproterozoic black shales support the idea of widespread Mesoproterozoic ocean anoxia and low atmospheric oxygen levels^9,10^. Furthermore, the low degrees of fractionation of sedimentary chromium^11^ have been used to suggest the lack of oxidative weathering of chromium minerals on land and thus the very low atmospheric oxygen concentrations of ≤0.1% of present atmospheric levels (PAL). However, other evidence, i.e., the abundance of 2,3,6-trimethyl aryl isoprenoids (2,3,6-TMAI) and the low concentrations of the trace element V in the marine black shales of Unit 3 of the Xiamaling Formation have been used to argue for an ancient oxygen-minimum zone (OMZ) with oxygenated bottom waters^12^. The further water-column modeling of carbon mineralization has provided estimates of high atmospheric oxygen levels of >4% PAL. Furthermore, Unit 1 of the Xiamaling Formation shows evidence of extensive organic matter mineralization, and the diagenetic modeling of these sediments has provided further evidence of elevated atmospheric oxygen levels of greater than 1% to 10%^13^. These estimates differ greatly from previous estimates of very low oxygen levels based on Cr isotopes^11,12,14,15^. Additionally, a study of Cr isotopes in 900 to 1100 Ma marine carbonates revealed highly fractionated isotopic compositions, indicating the oxidative weathering of Cr from land under relatively high atmospheric oxygen concentrations^16,17^. These greatly contrasting lines of evidence highlight the need for the further exploration of Mesoproterozoic-era marine oxygenation and the associated diagenesis of Mesoproterozoic organic matter.

Sedimentary lipid biomarkers have become important tools for the reconstruction of diagenesis in palaeo-environments, and rearranged hopanes have received increasing attention as biological markers with applications for the geochemical studies of petroleum source rocks and oils^18–21^. Although the precise pathways for the formation of rearranged hopanes remain unresolved, the depositional redox environment and lithology have been argued to be the critical factors for the rearrangement of hopanes^18–20^. In this paper, we focus on biomarker evidence, particularly the distribution of hopanes and rearranged hopanes, to further assess the redox conditions during the deposition of Units 2 and 3 of the Xiamaling Formation.

## Geologic Background

**Figure 1:**
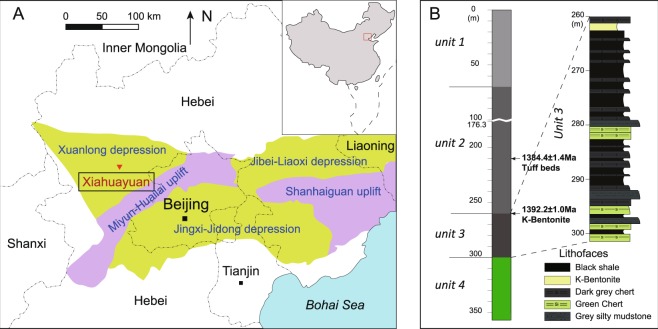
The distribution and lithological profile of the Xiamaling Formation of the North China Block (modified after Luo *et al*.^80^).

The Xiamaling Formation was deposited on the North China Block in a tropical to sub-tropical setting between the latitudes of 10°N and 30°N^22^ (Fig. 1A). The Xiamaling Formation features the prominent layering of alternating sediment types throughout most of its thickness, and its sedimentary lamination patterns are consistent with the influence of climate forcing on the sedimentary dynamics throughout much of the formation^23^. Thermal ionization mass spectrometry (TIMS) dating yielded ages of 1384.4 ± 1.4 Ma for a tuff layer 210 m below the top of the formation and 1392.2 ± 1.0 Ma for a bentonite layer 262 m below the top of the formation^23^. The Xiamaling Formation is inferred to have been deposited in a passive-margin setting before later back-arc development^24,25^.

The total thickness of the formation is approximately 450 m, and it can be divided into 6 Units (Fig. 1B). These highly laminated sediments were mostly deposited in deep, quiet waters below the storm wave base (>100 m)^26^. Unit 6 consists of cross-bedded sands and silts, with occasional ferruginous concretions; Unit 5 consists of brown marlstones interbedded with laminated and organic-poor silts, sands and muds; and Unit 4 is mostly composed of alternating red and green muds and green sandy silts, with occasional sand layers. Unit 3 begins with the appearance of black shale layers and ends with green muddy silts, while the middle part is characterized by alternating black shale and chert without any evidence of turbidite deposition or mass flows. Unit 2 consists of continuous black shales, which are interpreted to have been deposited in deep-water quiet depositional conditions, and Unit 1 is composed of alternating layers of black and green shales^13,23,26^.

**Figure 2:**
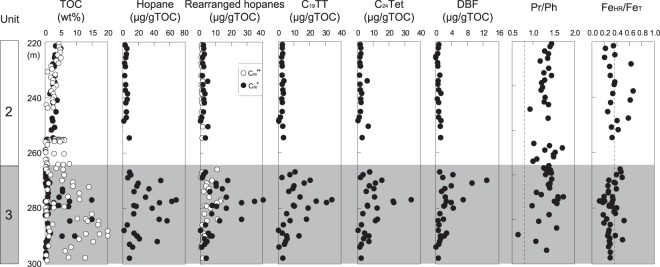
The TOC concentration, biomarker index, and Fe speciation in the OMZ (Unit 1) and outer OMZ (Unit 2) strata (TOC: total organic carbon; C_19_TT: C_19_-tricyclic terpanes; C_24_Tet: C_24_-tetracyclic terpanes; DBF: dibenzofuran; C_30_*: 17α(H)-diahopanes (C_27_ and C_29_-C_35_); C_30_**: C_30_ early-eluting rearranged hopanes; Fe_HR_/Fe_T_: highly reactive/total iron; see Supplementary material for a complete set of data).

Some of the geochemistry of the Xiamaling Formation has previously been described^12,13,26^. We focus here on the lower Unit 2 and, in particular, Unit 3. The shales of both Units are enriched in total carbon content (TOC)(Fig. 2), but Unit 3 is more enriched, with TOC values approaching 20 wt% in some cases. Unit 2 records simultaneous enrichments in vanadium (V), molybdenum (Mo) and uranium (U). These enrichments are found together with aryl isoprenoids^12,26^, which primarily represent the breakdown products of the aromatic C_40_ carotenoid isorenieratene, thereby highlighting the presence of sulphides or Fe-oxidizing green sulfur bacteria (BSBs) and photic-zone anoxia in the water column^27^. The ratios of highly reactive to total iron (Fe_HR_/Fe_T_) are both greater than and less than 0.38 in Unit 2 (Fig. 2). Ratios of Fe_HR_/Fe_T_ of >0.38 indicate deposition under anoxic conditions, but anoxic environments can also have Fe_HR_/Fe_T_ values of <0.38 if the sedimentation rate is rapid or, more generally, if the water column supply of highly reactive iron is low for whatever reason^28–30^. The iron enrichments and abundance of aryl isoprenoids indicate that Unit 2 was deposited under anoxic depositional conditions.

Although Unit 3 is TOC-rich compared with Unit 2, it displays very different geochemical characteristics. The sediments are enriched in Mo and U but generally depleted in V^12^. As previously explored^12^, such conditions are found in the oxygenated waters below the cores of modern oxygen-minimum zones (OMZs). Adding to this interpretation are the observed enrichments in aryl isoprenoids, which indicate the presence of sulphides or Fe-oxidizing green sulfur bacteria in the upper anoxic part of the water column^12^. As mentioned above, these observations have been used in ocean modeling to constrain atmospheric oxygen to >4% present oxygen levels (PAL)^12^.

Since the maturity of the organic matter in the Xiamaling Formation in the Xiahuayuan area (Fig. 1A) is low (equivalent vitrinite reflectance ~0.6%, Tmax <450 °C)^12^ and the TOC concentrations of the black shales are very high in Units 2 and 3, large quantities of well-preserved biomarker molecules are extractable. The high abundances of regular hopanes and rearranged hopanes were detected in the biomarker composition, thereby allowing the further analysis of the sedimentary redox conditions at the time of the deposition of the Xiamling Formation.

## Results and Discussion

### Organic composition patterns

**Figure 3:**
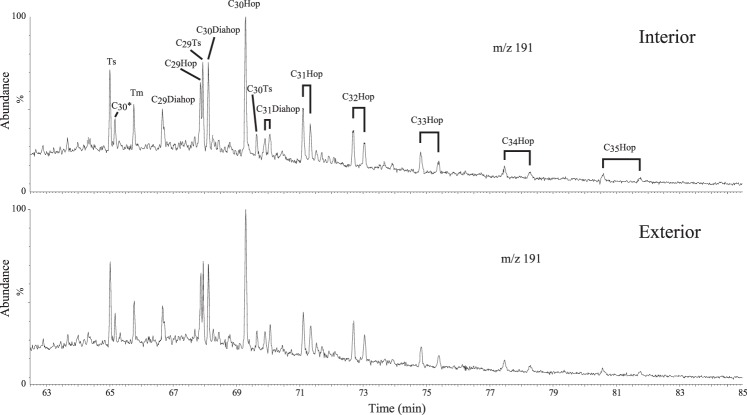
Partial m/z 191 mass chromatograms showing hopane distributions in the exterior surfaces and interior rock of sample at a depth of 272.5 m. Analyses were carried out at Australian National University (hop: hopane; diahop: diahopane; Ts:18a(H)-22,29,30-trisnorneohopane; Tm:17a(H)-22,29,30-trisnorhopane).

Biomarkers can often provide crucial information about the microbial ecosystem and environmental evolution, but the syngeneity of Precambrian biomarkers must be carefully evaluated^31^. We present evidence to support the syngeneity of molecular biomarkers in the Xiamaling Formation. First, the organic matter has experienced only low levels of thermal maturity, and the rocks have high TOC concentrations^23,32^, both of which are favorable conditions for preserving extractable biomarker molecules. Second, the biomarkers in the Xiamaling Formation black shales are distinctly different than from those in the overlying Jurassic coal-measure strata^12^. Thus, the Xiamaling Formation does not contain younger Jurassic biomarkers or any other demonstrable biomarkers from the Phanerozoic era. Third, the comparison of the biomarker results between the exterior surfaces and interior of a sample, carried out at Australian National University, reveals that the hopanes on the exterior surfaces and those in the interior of the rock are identical (Fig. 3). Thus, the hopanes of these Xiamaling Formation samples are interpreted to be indigenous and to not have been affected by any contamination during either the sampling or analytical procedures. Lastly, the Xiamaling Formation itself displays significant differences in its biomarker abundances and compositions at different depths^12^. For example, significant differences in the abundances of terpanes and hopanes exist between Unit 2 and Unit 3 (Fig. 2). Overall, the hopanes and terpanes in the Xiamaling sediments are interpreted to represent *in situ* organic signatures and to record information about the marine biological community at the time of the deposition of the Xiamaling Formation.

### Hopane distributions

**Figure 4:**
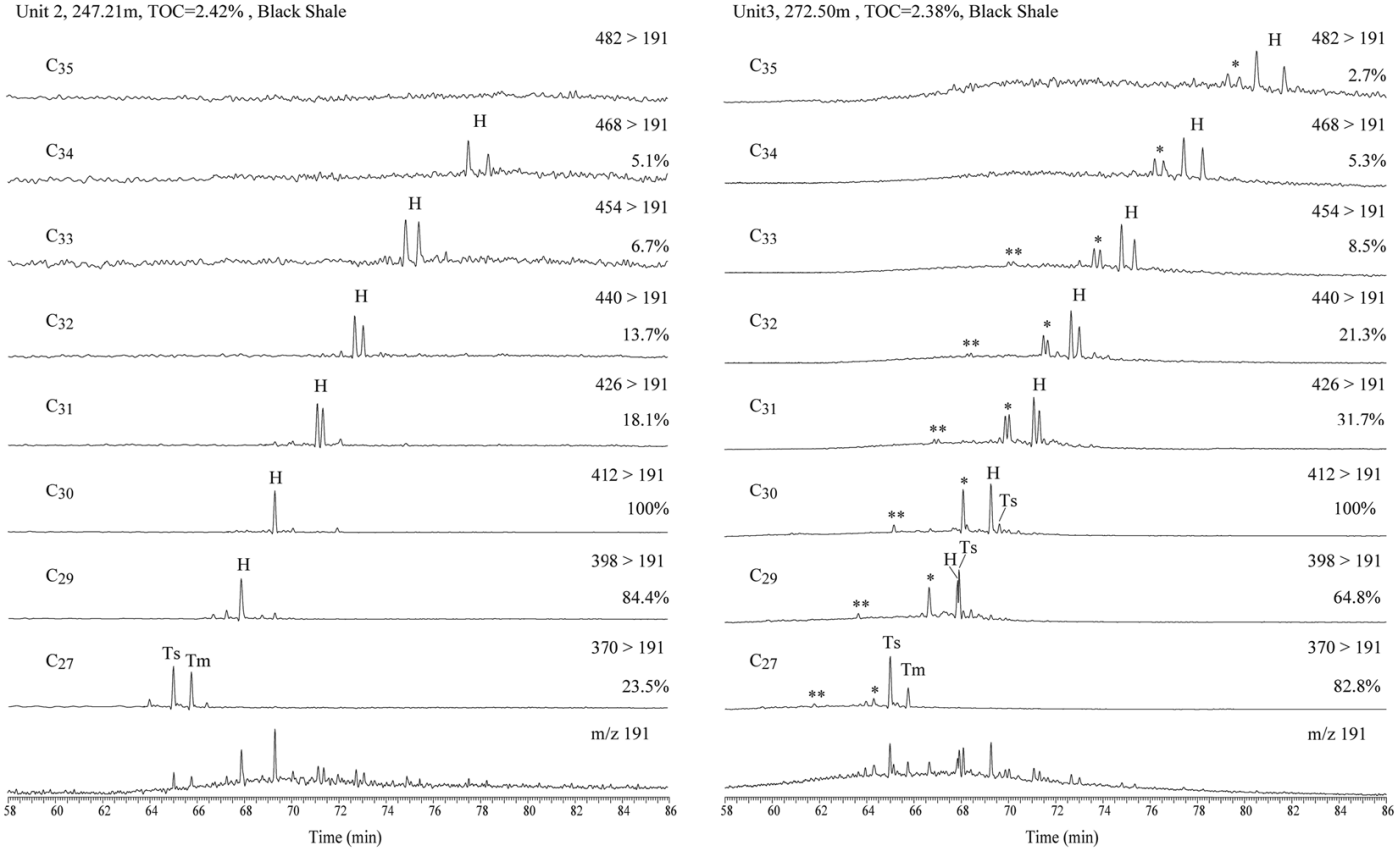
Partial m/z 191 mass chromatograms (bottom) and MRM data showing the C_27_ to C_35_ hopane and diahopane series in Unit 3 (272.5 m) and the overlying Unit 2 (247.21 m) (H: C_30_17α(H)-hopane; *17α(H)-diahopanes (C_27_ and C_29_-C_35_); **C_30_ early-eluting rearranged hopanes).

Hopane and terpane compounds were identified in the saturated fractions, with the fragment ion of m/z 191 as the base peak. A series of regular hopanes and rearranged hopanes were detected in the OMZ sediment of Unit 3, while there are almost no rearranged hopanes in the overlying Unit 2 (Figs 2 and 4). The three series of rearranged hopanes in Unit 3 include high concentrations of 17α(H)-diahopanes (C_27_ and C_29_-C_35_) (* or diahopanes) and 18α(H)-neo-hopanes (Ts, C_27_ and C_29_-C_30_), as well as another series of early-eluting rearranged hopanes (EER-H) (**) (Fig. 4). The early-eluting series has been known for a long time^19,33–38^, but its structure is currently undetermined, and it has only once been tentatively identified as 9,15-dimethyl-25,27-bisnorhopanes^39^. Compared with the composition of hopanes in the OMZ sediment of Unit 3, the overlying Unit 2 contains very little diahopanes or EER-H and even less C_29_Ts (Fig. 2). Previously, diahopanes have been detected in many sediments and petroleum^19,20,40^. Rearranged hopanes have been discovered in various sedimentary rock types from different time periods, including Phanerozoic terrestrial strata, such as Jurassic strata in the Sichuan Basin^41^ and Triassic and Tertiary lacustrine basins in China^18,21^, as well as in some Mesoproterozoic strata and in fluid-inclusion oil in Australia^42,43^. The rearranged hopanes are generally believed to be consistent with an origin characterized by catalytic rearrangement from hopenes during early diagenesis, and hopanes may have been derived from heterotrophic bacteria or cyanobacteria in the paleoecosystem during the deposition of its source rock^19^. However, these rearranged hopanes are seldom connected with the redox environment.

The peak concentrations of C_30_* and C_30_** are found in Unit 3 at depths of 290–270 m and represent 40 μg/g TOC and 12 μg/g TOC, respectively. Such high concentrations of C_30_* have previously been interpreted to be indicative of plant material derived from terrestrial strata^44^, but the input of terrestrial material during the Mesoproterozoic is obviously impossible. Additionally, the concentrations of C_19_TT and C_24_Tet are also very high in this interval, i.e., significantly higher than those in the upper strata (Fig. 2). The Pr/Ph ratios range from 0.6~1.6, and the ratio is slightly higher in Unit 3 than in Unit 2. High Pr/Ph ratios (>3.0) indicate the input of terrigenous organic matter under oxic conditions, while low values (<0.8) typify anoxic, commonly hypersaline or carbonate environments. However, the Pr/Ph values of Xiamaling, which fall within a fairly narrow range (0.8–3), are still influenced by many factors in addition to depositional redox conditions^45,46^. The origins of pristane and phytane are believed to originate from many complex mechanisms other than the reduction or oxidation of the phytol side chain in chlorophylls. They are even formed by the thermal cleavage of isoprenoid moieties bound by non-hydrolyzable C–C and/or C–O bonds within the kerogen matrix^45^. Goosens *et al*. suggested that tocopherols could also be a major source of pristine^47^. Methyltrimethyltridecylchromans (MTTCs) are regarded as another alternative source of pristane and phytane, and MTTCs have been proposed to have a biological origin^48^. Therefore, it is difficult to determine depositional redox conditions using only the Pr/Ph ratio without the support of another index.

Additionally, there are trace C_35_ hopanes at m/z 191 (Fig. 3); the absolute concentration of C_35_ hopanes is very low, just 2.7% of C_30_ hopane, and about half the abundance of the C_34_ homohopanes. While high C_35_ homohopanes are interpreted as a general indicator of highly reducing marine conditions during deposition^49^, high C_35_ hopane ratios for extracts are also correlated with high hydrogen indices in the source rocks due to the better preservation of oil-prone organic matter^50^. In addition, the homohopane index has been observed to decrease with maturity in a suite of related oils derived from the Monterey Formation, California, indicating that homohopane distributions are also affected by thermal maturity^51^. Therefore, it is also difficult to determine the redox conditions based on the trace C_35_ hopanes in the Xiamaling sediments.

### The precursor of hopanes and rearranged hopanes

Hopanoids are generally believed to be derived from a wide range of bacteria and bacteria-generated products, such as bacteriohopane and diplopterol, which play important functions in bacterial cell membranes, depending on the cell form and size. These compounds are the structural equivalents of the sterols found in eukaryotes^52^. Their importance as membrane constituents is responsible for their common occurrence in hydrocarbons and petroleum derivatives, especially those from the early Paleozoic era (or even the Precambrian), prior to the development of higher plants^53^. Bacteriohopanoids have been detected in some, but not all, cyanobacteria^54^, purple non-sulfur bacteria, and gram-negative and gram-positive bacteria^55^.

Where, then, do the rearranged hopanes come from? The carbon skeletons of these rearranged hopanes have never been detected in bacteria or plants, and they are different from those of known bacterial hopanoids. An early study reported that rearranged hopanes probably arise through chemical mechanisms occurring during the diagenesis of natural non-rearranged hopanoid products^19^. Moldowan *et al*.^19^ determined that hopanes and various rearranged hopanes are isotopically very similar, suggesting that they are genetically related^19^. Thus, the existence of high concentrations of rearranged hopanes, combined with the high concentrations of regular hopanes, illustrates the possible prominent contributions of bacteria in Unit 3 of the Xiamaling Formation.

The exact mechanism by which hopanes change into rearranged hopanes remains debated^41,56^, including the initial viewpoint that rearranged hopanes are derived from terrigenous sources^44^. Rearranged hopanes have also been attributed to the catalytic rearrangement of regular bacterial hopanoids by clay minerals (Moldowan *et al*., 1991), and they have also been assumed to reflect high maturity according to the thermodynamic stability values calculated by molecular mechanics^57^. The C_30_* series is considerably more stable than the C_30_ regular hopane series and 18α(H)-neohopane series^57^; thus, the C_30_ series is a useful maturity parameter at even higher maturities, according to Molowan^19^. Thus, the C_30_*/(C_30_* + C_30_) ratio, along with sterane isomerization in oils, has been used to map maturity gradients in the North Sea oil fields^58,59^. In contrast, due to the similarly low degrees of thermal maturity of all the Xiamaling sediments^23^, maturity can be excluded as a major control of the observed rearranged hopane distribution; thus, the transition mechanism from hopanes to rearranged hopanes is unlikely to be related to thermal alteration.

Based on the similarity of the C_30_*, C_30_** series and diasteranes in Toarcian samples, it has been suggested that the acid (clay)-catalyzed “backbone rearrangement”, with the participation of oxygen, may be responsible for the diagenetic formation of the rearranged skeleton structures of hopanes and steranes^20^. This process is analogous to the formation mechanism of diasteranes from sterane precursors. However, the acid (clay) catalysis hypothesis remains controversial^60^ because if diahopanes are formed via shifts in the carbon skeleton in the same way that diasteranes are formed from sterane precursors, the rearrangement of diploptene (or hop-17(21)-ene) should lead to the formation of neohopene, fernene, adianene and ultimately filicene structures, but not diahopane^20^.

Plant triterpenoid biosynthesis could also lead to such a rearrangement^61^, but under normal clay catalysis conditions, except for neohop-13(18)-ene, this rearrangement process could not proceed^60^. In this case, rearranged hopanes could not be the direct result of acid (clay)-catalyzed products because in natural digenesis, double-bond isomerizations via secondary carbocations are thermodynamically unlikely to occur based on molecular mechanics^62^. We note that the samples from Unit 3 of the Xiamaling Formation OMZ are all black shales, not the different samples of black shales and carbonate or marl discussed by Luo^32^. Additionally, Unit 2, similar to Unit 3, also consists of black shale; however, in contrast, it has a very low content of rearranged hopanes. Therefore, the very different concentrations of rearranged hopanes in Unit 2 and Unit 3 do not support the hypothesis that the formation of rearranged hopanes is related to the catalysis of clay minerals. Overall, the rearranged hopanes in the Xiamaling Formation did not result from thermal maturity or acid (clay)-catalysis, and they were not sourced from terrigenous sources.

The rearranged hopane structure has also been suggested to form by the oxidation of the C-l6 alkyl position of 17α(H)-hopanes, which results in the subsequent formation of a δ^15,16^ double bond and the rearrangement of the methyl group from C-14 to C-15^19^. Therefore, we argue that the rearranged hopanes in Unit 3 were more likely to have formed through chemical mechanisms as an allylic selective oxidation step at C-16 from unrearranged hopanoid natural products during oxic diagenesis. It appears that the formation of rearranged hopanes is best interpreted to involve the participation of oxygen. As noted above, there would have been some oxygen in the sediment-water interface, thus supporting the occurrence of aerobic diagenesis during the deposition of Unit 3.

### The redox significance of rearranged hopanes

Considerable evidence from Phanerozoic-aged environments has shown that rearranged hopanes can be associated with terrestrial facies. For example, C_30_* was first detected in Upper Jurassic rock extracts and crude oil from the Barrow sub-basin, Western Australia, by Volkman *et al*.^63^. Later, Philp and Gilbert also found C_30_* in many terrestrial facies, such as those in the Gippsland Basin, Sydney Basin, Cooper/Eromanga Basin and Surat/Bowen Basin^44^. High concentrations of rearranged hopanes were also reported in terrestrial lacustrine sediments in the Songliao Basin and Ordos Basin^18,64^. These compounds coexist with high concentrations of C_24_ Tet and 18α(H)-oleanane, which have been discovered in Tertiary terrestrial crude oil and are consistent with the presence of angiosperms. Thus, rearranged hopanes have been considered to be generated along with the biomarker of terrestrial biomass; therefore, rearranged hopanes have been assigned to terrestrial environments^33,44,65^. In addition, generally rearranged hopanes and their coupling with depositional environments have most commonly been explored in the Phanerozoic era.

Not all terrestrial lacustrine sediments in the Ordos Basin, however, are rich in rearranged hopanes. For example, while the concentration of C_30_* is very high in the dark mudstones of the Chang 4 + 5 to Chang 9 members of the Upper Triassic Yanchang Formation in the Ordos Basin, China, a portion of the Chang 7 Member has very low C_30_* concentrations^18^. Based on their very low U/Th and V/Sc ratios, low sulfur contents and depositional characteristics, the Chang 4 + 5 to Chang 9 members were interpreted to have been deposited in an oxic shallow lake or semi-deep lake environment in the Ordos Basin, China^18^. In contrast, the Chang 7 Member was likely deposited in a deep anoxic lake environment, and it has very low C_30_* concentrations, high U/Th and V/Sc ratios, and high sulfur contents^18^. Therefore, these observations suggest that the redox environment can greatly influence the abundance of C_30_* and that oxic conditions are favorable for the formation of C_30_*. Therefore, the relative content of C_30_* has been suggested to be an environmental index for lacustrine sources (oil shale and dark mudstones), where high abundances of C_30_* indicate oxic environments of shallow to semi-deep lacustrine facies^18,66^. The anomalously high concentrations of rearranged hopanes in upper Paleozoic coal sediments were also attributed to an oxidizing sedimentary environment, as confirmed by the parameters of methylphenanthrenes^66^. The Jurassic oil generated from lacustrine sediments in the Sichuan Basin, which were deposited in an oxidizing environment, also have high C_30_* contents (>>C_30_ hopane)^41^.

Asif *et al*.^67^ also discovered that Class A crude oil, with very high ratios of C_30_*/C_30_ αβ and a pristane (Pr)/phytane (Ph) ratio of 3.2, was formed from terrestrial organic matter in a highly oxygenated river delta environment (i.e., a highly oxic depositional environment) in the Potwar Basin in Pakistan. In this case, high Pr/Ph values were argued to have been formed by the input of terrestrial plant material into oxidizing or weakly oxidizing conditions^45,68,69^. The Class A crude oil in the Potwar Basin in Pakistan is also rich in C_19_TT, C_24_Tet, and dibenzofuran (DBF). C_19_TT and C_24_Tet are also believed to be associated with a predominantly terrigenous organic matter source^45,70,71^. Asif *et al*.^72^ found that by adding molecular oxygen, sulfur and nitrogen compounds to biphenyl (BP), large quantities of DBF, dibenzothiophene (DBT) and carbazole were produced due to the effects of activated carbon, and the resulting compounds formed in proportion to the amounts of O, S and N in the original kerogen. Hence, C_19_TT and C_24_Tet, DBF and rearranged hopanes are all assumed to form in oxidizing environments based on the experimental analysis of Class A crude oil.

By analogy to the high concentrations of rearranged hopanes and high contents of C_19_TT and C_24_Tet in the oxygenated terrestrial depositional environments discussed above, we argue that the rearranged hopanes in Unit 3 of the Xiamaling Formation were formed by the rearrangement of the methyl group from C-14 to C-15 in the presence of oxygen^19^.

The Xiamaling Formation is not the only Mesoproterozoic stratum in which rearranged hopanes have been discovered^43,53^. Blumenberg *et al*.^73^ reported different ratios of C_30_-diahopane and C_24_Tet in the low-maturity (Rc ~ 0.47–0.56) black shales of the Touirist Formation, northwestern Africa. The Touirist Formation was regarded as having formed in a shallow-water setting with the potential for the diffusion of oxygen into the upper water column from the atmosphere or from the effects of episodic storm mixing due to the lack of extensive euxinic conditions in the water column during the deposition of the Touirist Formation based on its observed low aryl isoprenoids and high C/S-ratios^73^. Therefore, the high concentration of C_30_* was also believed to have been produced by the participation of oxygen in the Mesoproterozoic Touirist Formation.

The rearranged hopanes reported in fluid inclusions in an igneous intrusion and organic-rich sediments in the Middle Proterozoic McArthur Basin, Northern Australia^42,74^, as well as the dolostone from the Late Proterozoic Walcott Member, Chuar Group, Grand Canyon, Arizona^53^, did not directly bind the hopanes to the redox condition under which they formed. In this case, it is difficult to interpret the environmental significance of the rearranged hopanes.

Overall, although rearranged hopanes in Mesoproterozoic sediments have been reported, few studies have connected the occurrence of rearranged hopane to their oxygen levels and resultant diagenesis. The Phanerozoic rearranged hopanes provide a good analogy for the aerobic diagenesis of organic matter in the Xiamaling Formation interval, or even the oxic Mesoproterozoic.

### The prerequisite oxygen level for aerobic digenesis

**Figure 5:**
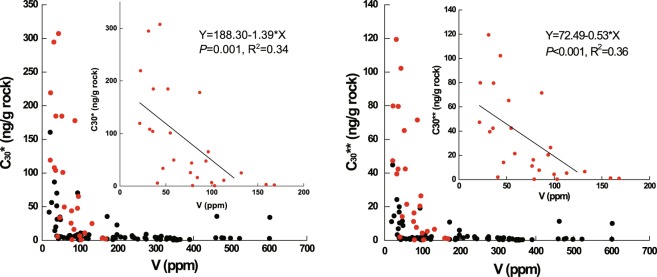
The negative correlation between V and rearranged hopanes (outcrop samples: black dots; core samples: red dots; C_30_*: 17α(H)-diahopanes (C_27_ and C_29_-C_35_); C_30_**: C_30_ early-eluting rearranged hopanes).

The mineralization of organic matter can occur under the conditions of aerobic or anaerobic microbial activity depending on bottom-water O_2_ concentrations. The high concentration of rearranged hopanes suggests that the aerobic diagenesis of organic matter occurred in Unit 3 of the Xiamaling Formation. Based on the negative correlation observed between V and rearranged hopanes (Fig. 5), we can further constrain the occurrence of aerobic diagenesis at the water-sediment surface. This is because V is typically not concentrated (and is sometimes even released) from sediments deposited under low-oxygen (but still oxygenated) conditions and sometimes under normal bottom-water oxygen levels where oxygen only penetrates a few mm into the sediment^12^. Therefore, low sediment V concentrations directly indicate an oxic water-sediment interface^75^. Thus, the negative correlation between V and rearranged hopanes suggests that more oxic conditions result in sediments with lower V contents and higher contents of rearranged hopanes. This further supports our conclusion that sediments with high contents of rearranged hopanes likely formed under oxic conditions.

We conclude that the high concentration of rearranged hopanes in Unit 3 of the Xiamaling Formation marine sediments is most likely connected to an oxygenated depositional environment. However, the degree of oxygenation depends on the oxygen available in the ancient biogeochemical cycle. During the Phanerozoic era, oxygen was widespread throughout most of the ocean and likely permeated marine sediments to depths ranging from a few millimeters to several centimeters^76^. It is likely that atmospheric oxygen levels were much lower during the Mesoproterozoic era. Some have suggested an atmospheric oxygen content of less than 0.1% PAL^11^. At these levels, oxygen would likely have been unavailable to penetrate into marine sediments, particularly organic-rich sediments, such as those deposited in Unit 3 of the Xiamaling Formation. Therefore, the high concentrations of rearranged hopanes point to higher levels of atmospheric oxygen, supporting the values of >4% PAL determined by Zhang *et al*.^12^ for Unit 3 of the Xiamaling Formation and 3–10% PAL determined^12^ for Unit 1 of the Xiamaling Formation^13^. In addition, the discovery of large, up to 30-cm-long and nearly 8-cm-wide multicellular eukaryotes from the 1.56-billion-year-old Gaoyuzhuang Formation in North China^77,78^ could further indicate biological respiratory demands in excess of what <0.1% PAL could provide. Altogether, elevated oxygen levels of >4% PAL are likely more consistent with the requirement that the surface sediment of Unit 3 contained enough oxygen for the hopane precursors to transform into rearranged hopanes during the early stages of diagenesis. In contrast, the anoxic bottom-water conditions of Unit 2 clearly lacked the oxygen for this transition^12,26^.

## Conclusions

High concentrations of various rearranged hopanes were identified in the saturated fraction of the organic-rich Unit 3 of the Xiamaling Formation of North China. Based on considerable Phanerozoic terrestrial evidence, the high concentrations of rearranged hopanes are inferred to have formed from hopane precursors in an oxygenated environment. We propose that benthic aerobic microbial activity was active during the Mesoproterozoic era and that this activity influenced the early diagenetic evolution of primary biomarkers, leading to high contents of rearranged hopanes. These processes were very similar to those that occurred in oxygen-rich Phanerozoic sedimentary environments. The presence of Mesoproterozoic rearranged hopanes points to an aerobic sedimentation environment during the Mesoproterozoic.

## Material and Methods

### Samples

All of the samples were collected from a core using a diamond drill lubricated with fresh water to minimize contamination from drilling fluids^23^. For the geochemical analyses, all samples were rinsed with purified water, dried, and then crushed into fine powders (less than 74 μm) using a stainless steel puck mill, which was cleaned between samples by grinding baked quartz sand multiple times.

### Trace element analysis

The V concentrations were measured via inductively coupled plasma mass spectrometry (ICP-MS) following the methods outlined in^23^. Accuracy was tested using the shale standard GBW 03014, which was measured along with the samples, and the results were determined to be within 3.0% of the accepted value of V. The concentrations of Al were measured using X-ray fluorescence. The accuracy was tested relative to the whole-rock standard materials GBW 07109–07112, and the relative standard deviation of major element concentrations was <1%.

### Biomarker extraction and analysis

The rock samples were crushed to a grain size of 100 mesh. All of the glass vessels used for bitumen extraction were combusted at 700 °C in a muffle furnace to remove any organics and ultrasonically washed with purified water. Then, 200 g of precisely weighed powder was extracted using Soxhlet extraction with chloroform for 8 h. The extracts (chloroform bitumen “A”) were kept in beakers (100 ml) until the chloroform completely evaporated in a fume hood. Then, 25 mg of bitumen “A” was dissolved in an appropriate amount of n-hexane, and the internal standards D_10_-anthracene, 5α-androstane and C_24_D_50_ were added. Approximately 12 hours later, the asphaltene precipitate was separated by filtration, and the filtrate was divided into saturated hydrocarbons, aromatic hydrocarbons and polar fractions using 10 ml hexane followed by 20 ml dichloromethane:n-hexane (2:1) as eluents in a silica gel glass column (100~200 mesh, activated at 200 °C for 4 h).

The gas chromatography mass spectrometry (GC-MS) analyses of the hydrocarbon fractions were performed using a Thermo Scientific TRACE GC Ultra-DSQ II mass spectrometer. An HP-5 chromatographic column (60 m × 0.25 mm × 0.25 µm) was used to separate the saturated and aromatic hydrocarbon fractions. For the saturated hydrocarbons, the oven temperature was initially set at 70 °C for 5 min and was programmed to increase at 4 °C/min to 220 °C followed by 2 °C/min to 320 °C, where it was held for 20 min. For the aromatic hydrocarbons, the oven temperature was programmed to remain at 70 °C for 5 min, increase to 320 °C at 3 °C/min increments, and then remain isothermal for 20 min. Helium was used as a carrier gas, with a constant flow rate of 1 ml/min. Both the interface temperature and the injection temperature were 300 °C. The transfer line temperature was 250 °C, and the ion source temperature was 230 °C. The ion source was operated in electron impact (EI) mode at 70 eV, and selected ion monitoring (SIM) was performed.

To verify the indigenous biomarkers of rock samples, the differences between the biomarkers of the exterior surfaces and interior rock were analyzed at Australian National University. In addition, the exterior surfaces and interior rocks at a depth of 272.5 m are displayed in Fig. 2. The entire surfaces of samples were cut using a clean precision diamond blade (with a thickness of 0.85 mm) according to Brocks^79^. The combined surfaces of the samples and the remaining cores were separately crushed into powder, extracted and fractionated as described above for the analysis of bitumen “A”. Then, GC-MS analyses were carried out to obtain the biomarker patterns of the hopane series.

## Electronic supplementary material

Supplementary Dataset
